# A New Proximal Femur Reconstruction Technique after Bone Tumor Resection in a Very Small Patient: An Exemplificative Case

**DOI:** 10.3390/children8060442

**Published:** 2021-05-25

**Authors:** Carmine Zoccali, Silvia Careri, Dario Attala, Michela Florio, Giuseppe Maria Milano, Marco Giordano

**Affiliations:** 1 Oncological Orthopaedics Department, IRCCS—Regina Elena National Cancer Institute, Via Elio Chianesi 53, 00144 Rome, Italy; carmine.zoccali@ifo.gov.it; 2 Department of Orthopaedics and Traumatology, Bambino Gesù Children’s Hospital, IRCCS, Piazza di Sant’Onofrio 4, 00165 Rome, Italy; michela.florio@opbg.net (M.F.); marco.giordano@opbg.net (M.G.); 3 Muscular-Skeletal Tissue Bank–IRCCS–Regina Elena National Cancer Institute, Via Elio Chianesi 53, 00144 Rome, Italy; dario.attala@ifo.gov.it; 4 Department of Pediatric Hematology/Oncology and Stem Cell Transplantation, Bambino Gesù Children’s Hospital, IRCCS, Piazza di Sant’Onofrio 4, 00165 Rome, Italy; giuseppemaria.milano@opbg.net

**Keywords:** bone tumors, Ewing’s sarcoma, infants, children, composite prosthesis

## Abstract

For patients too young to be fitted with an expandable prosthesis, limb salvage surgery requires other strategies. The main problems are related to the impossibility of implanting an expandable prosthesis to the residual bone growth that is much too big in relation to the bone size, with the precocious implant loosening and/or the residual absence of bone growth, as well as the problem of limb length and shape difference. In this paper, we report a possible reconstruction solution using a composite prosthesis for an Ewing’s sarcoma of the proximal femur in an infant patient. After resection, a femoral stem was cemented into the distal third of a homoplastic humerus; a carbon fiber plate was used to stabilize the bone/homograft interface. At the one-year follow-up, the patient was free of disease and able to walk with only a slight limp. This case report describes a possible solution for very small patients. An adult humerus is of the right size to replace a child’s lower limb segments, and the distal humerus can be shaped, maintaining a cortex stiff enough to support a prosthesis. Very young patients might obtain a faster osteointegration of the graft than adults, due to their higher biological activity and, in this case, the diapasonal shape of the allograft might also have contributed to accelerated fusion. Moreover, the use of a graft to fit the prosthesis avoids loosening issues due to canal widening, hypothetically providing more growing time before system failure and revision surgery. However, although this technique is promising, further studies are necessary to confirm our findings and to verify if this procedure allows easier future prosthesis implantation.

## 1. Background

Osteosarcoma and Ewing’s sarcoma represent the most frequent malignant primary bone tumors occurring in skeletally immature patients. While osteosarcoma is more common in the second decade of life, predominantly affecting the knee, Ewing’s sarcoma is also frequent in the first decade of life [1]. Treatments are multidisciplinary, and based on a combination of chemotherapy, radiotherapy and surgery [2]. Previously, amputation was considered the principal surgical treatment assuring a radical margin; however, improved chemotherapy schedules permit sparing the limb and increased survival [3,4].

Today, limb salvage surgery is considered the standard treatment for malignant tumors [5]. After bone tumor resection, the use of a megaprosthesis is considered the most common method of reconstruction [6]. This is true for adults and for fully grown patients; for growing patients, expandable prostheses can be a solution as long as the size of the bone segment permits the insertion of such a voluminous device [7,8].

In very small patients, in whom a prosthesis cannot be used, other strategies must be applied, and bone reconstruction becomes a challenging problem. Indeed, children have a different anatomy, so both resection and reconstruction are more difficult than in adults; moreover, modular prostheses are not commercially available in the required small sizes.

In fact, the main problems are related to bone growth, which could cause a precocious implant to mobilize due to the increasing bone diameter [9] and, obviously, the leg length discrepancy [10].

Possible solutions are as follows.

(1) Expandable prosthesis: this can be used only in older children where the residual bone is sufficiently long for a prosthesis with an elongation system to be inserted. Expandable prostheses are potentially able to compensate for the bone shortening after tumor resection, but results are poor due to loosening and breakage [9].
(2) Custom-made prosthesis: this is considered the most common treatment when modular prostheses are not available. Their use is very common in children. Custom-made prostheses can provoke aseptic loosening, and cause loss of bone stock, making revision surgery difficult and obtaining low functional results [11].
(3) Vascularized fibular flap: this technique was proposed by Manfrini et al., who replaced the femur by modeling the autogenous fibula, reproducing the femoral shape. The fibular epiphysis was used to imitate the femoral head [12]. The advantage of this technique is the potential growth of the fibula and its remodeling; nevertheless, it is a very difficult technique, and the success rate is quite low. Indeed, there are no case series in the literature.
(4) Extracorporeal irradiated autograft: this technique consists of reimplantation of the resected specimen after irradiation and soft tissue removal. It has the advantage of a perfect anatomical correspondence, although non-union is common [13]; moreover, it does not furnish complete information about histology and tumor necrosis after neoadjuvant chemotherapy. Although the hypothetical risk of local recurrence is present, it seems similar to that of other techniques which do not include the reimplantation of the specimen [14].
(5) Osteoarticular homograft: this is rarely used in isolation for the inferior limb, principally because it collapses under body weight and cannot articulate with the acetabulum, undergoing precocious resorption [15]. Moreover, these are not available for children, due to the absence of donors.
(6) Composite prosthesis (association of a joint prosthesis and a cemented homograft): this has the advantage of increasing the bone stock [16]; the prosthetic component should also guarantee a good articular motion. The corresponding homograft segment would be the best solution, but unfortunately, it is impossible to have child donors, so homografts from adult donors have to be adapted for young patients [10]. The homograft medullary canal is completely filled with cement in order to obtain higher resistance in weight-bearing.

In this paper we report a possible solution for reconstruction of the proximal femur in infant patients using a composite prosthesis; an exemplificative case is reported.

## 2. Case Report

**Figure 1:**
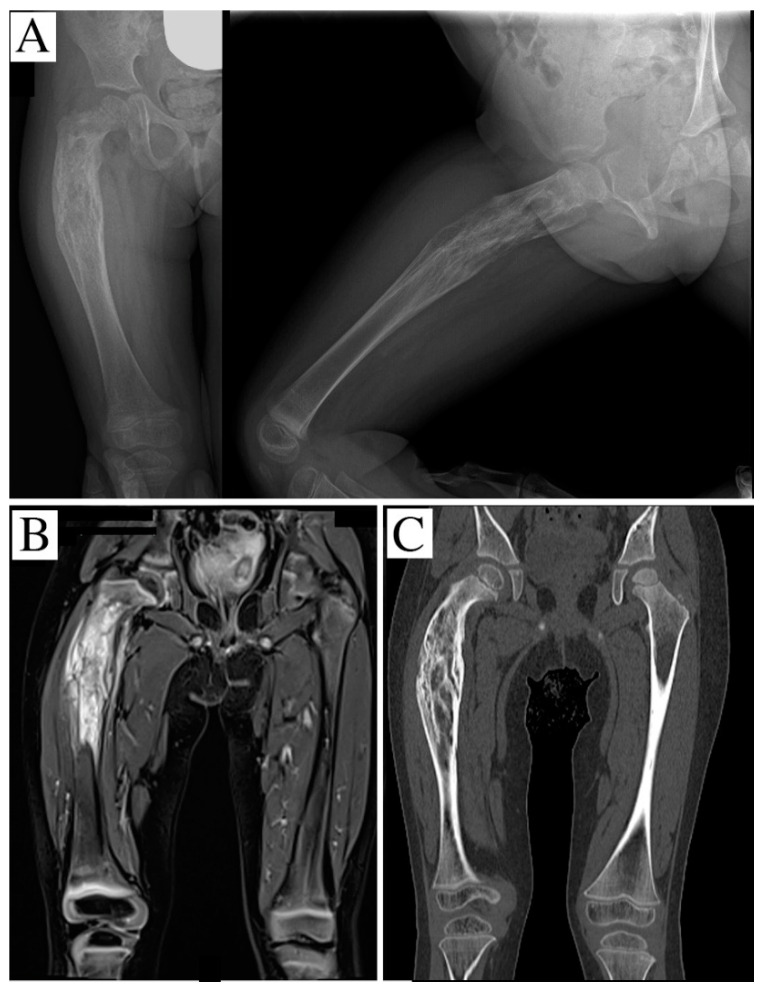
(**A**) an X-ray showing an osteolytic and apparently well-defined lesion of the proximal and middle third of the right femur; the segment is bent with onion-skin periosteal reaction; (**B**) STIR-weighted coronal-MRI reconstruction, evidencing permeative behavior and an intense edema. (**C**) CT scan showing the geographic osteolysis.

A girl aged 2 years and 8 months presented with the presence of a mass, indicative of malignancy, in her right proximal femur, and complained of continuous pain and limping. An X-ray (Figure 1A), MRI, scintigraphy and a CT scan were performed, and showed an osteolytic lesion in the proximal and middle femur. The bone cortex was extensively disrupted, but the proximal and distal femur growth cartilage was free from disease. The soft tissues mainly involved were the thigh anterior side muscles and, to a lesser extent, tissues in the posteromedial area, with the bone section globally embedded in the neoplasm; no metastases were detected, so a CT-trocar biopsy was performed. Histology revealed an Ewing’s sarcoma, and the patient was administered neoadjuvant chemotherapy with vincristine, cyclophosphamide, and doxorubicin. The subsequent CT scan and MRI, performed for surgical planning, showed multiple osteolytic areas beginning 8 mm from the growth plate and extending for 80 mm from the femoral neck base (Figure 1B,C). The distal edge of the sarcoma was 63 mm from the distal physis.

**Figure 2:**
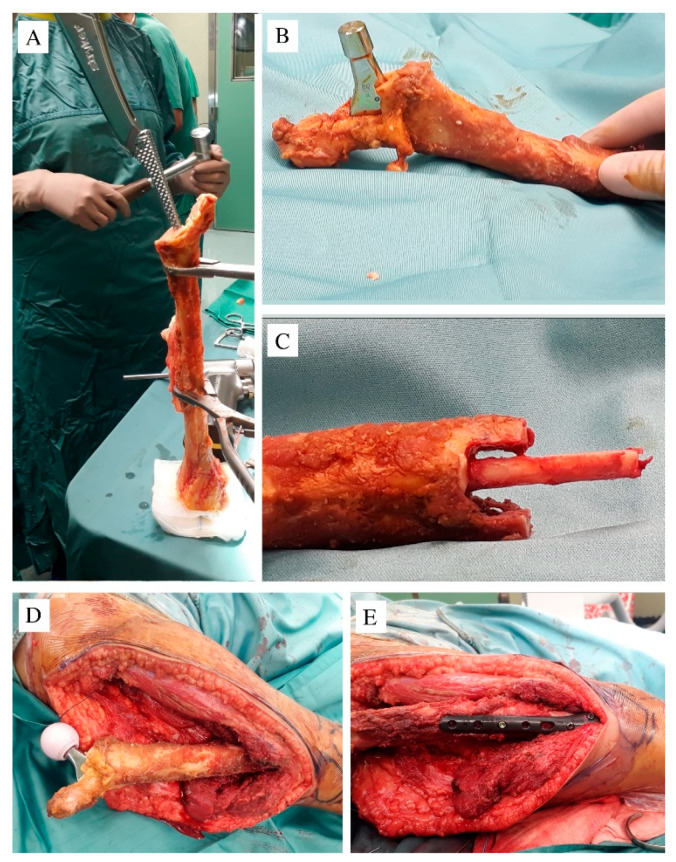
(**A**) The distal humerus after cutting of the medial epicondyle; the broaches were used to create a housing for the prosthesis; (**B**) the prosthesis cemented inside the canal; (**C**) the distal allograft was cut to a diapason shape to fit together with the patient’s residual femur; a cortical strut was cemented in the allograft medullary canal to increase the primary stability when inserted in the residual distal femur; (**D**) a ceramic head of 28 mm was applied; (**E**) the carbon fiber plate and screws used to stabilize the contact area between the allograft and the patient’s femur.

Surgery was performed in a specialized research hospital by a surgical team trained in musculoskeletal oncology, with more than 15 years’ experience of exclusively performing musculoskeletal tumor operations, and an experienced pediatric orthopedic surgeon. With the patient in a left lateral position, a direct lateral approach was performed; the proximal and middle femur was isolated, maintaining a layer of muscles to guarantee a wide margin. A femoral osteotomy was performed at 14 cm from the greater trochanter, and the proximal femur was removed after capsulotomy and sectioning of the rounded ligament. A distal section of a homoplastic humerus was then used for reconstruction. The allograft was obtained from “Regione Lazio Muscular Skeletal Tissue Bank”. It was stored frozen at −80 °C and not irradiated; bacteriologic and viral analyses were performed (with negative results), and before surgery, it was soaked in a Rifampicin antibiotic solution. The humeral medial epicondyle was removed; the olecranon fossa was drilled to access the medullary canal, and an adequate site for the implant was obtained using broaches (Figure 2A); then, a femoral stem prosthesis was cemented (Exeter DDH—Stryker, Kalamazoo, MI, USA) (Figure 2B). To increase the fusion, a diapason-shaped osteotomy was done in the distal extremity of the homograft and a cortical strut was placed and cemented inside to maintain the axis (Figure 2C).

A 28 mm ceramic femoral head was applied, the construct was connected to the residual distal femur (Figure 2D) and distally stabilized with a carbon fiber plate and screws (CarboFix Orthopedics Ltd., carbon fiber plate, Herzeliya, Israel) (Figure 2E). A capsuloplasty was done to decrease the risk of dislocation, and the gluteus muscles were stitched to the residual epicondyle, mimicking the great trochanter to the residual soft tissue. A transfusion of 300 mL of erythrocytes was administered during surgery, due to intra-operative blood loss.

A single hip spica was applied for 40 days and was then replaced by a thigh–foot half-cast in order to begin hip movement while still protecting the graft-to-host junction. After 30 days, the cast was removed. The knee extension was complete, but the maximum flexion was limited to 30°. Physical therapy started, focusing on range of movement recovery of the knee, but still maintaining no weight-bearing for another 30 days. The postoperative histology evidenced the presence of a macroscopic viable tumor (necrosis inferior to 70%), grade I responder according to Picci et al. [17]; the surgical margin was wide, so adjuvant radiotherapy was not indicated. In the meantime, adjuvant chemotherapy with vincristine, cyclophosphamide and doxorubicin was administered.

**Figure 3:**
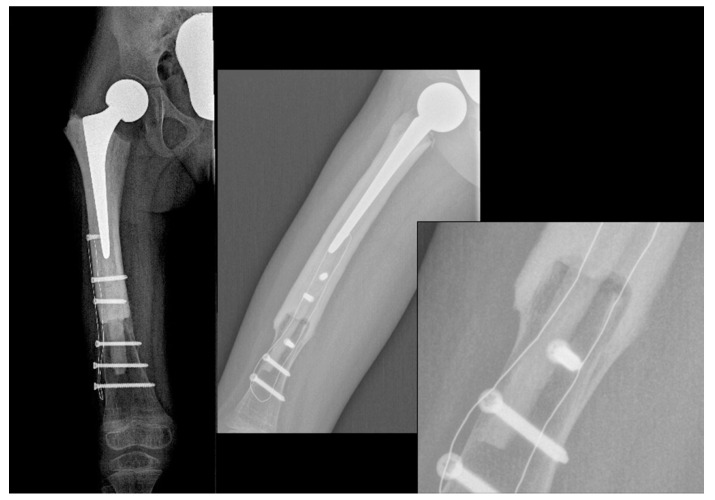
X-ray showing a stable fusion between the allograft and the femur.

At the one-year follow-up, the patient is apparently free of disease, and the allograft is completely osteointegrated (Figure 3). The young patient is able to walk with only a slight limp, as shown in Video S1 (link in Supplementary Materials). The right femur has been lengthened by 10 mm during surgery in order to reduce the leg length difference that will develop, and a 1-cm right shoe lift has been prescribed to obtain a temporary correction. Compared to the X-ray taken immediately after surgery, at the last follow-up, the distal femur had already grown 12 mm.

The active hip range of motion (ROM) was flexion 80°, extension 10°, abduction 20°. The active knee joint ROM was: flexion 110° and extension 0°.

This study was conducted in accordance with the World Medical Association Declaration of Helsinki of 1975, as revised in 1983, and the patient’s parents signed an informed consent form to allow the use of clinical data for research purposes and for publication. The local Ethics Committee approved the study (protocol number: 418/2021).

## 3. Discussion

Limb salvage surgery must be the goal to pursue when a wide resection of a tumor can be achieved. Thanks to sarcoma treatment improvement, the necessity for amputation has been reduced, but it maintains a fundamental role in certain cases [18]. At the same time, rotationplasty remains a viable option when limb-salvage is contraindicated [19], or if the distal femoral growth plate has to be sacrificed with consequent major limb length discrepancy [20]. The distal femoral growth plate of the patient was free from disease, and it was planned to spare it during surgery. As is known, distal femoral growth provides 70% of the bone length [21], thus, the use of the presented composite solution will allow the femur to grow to a near-normal length and width during the child’s growing up. Moreover, preoperative examinations of our patient showed no involvement of the main vessels or nerves, no metastases, and adequate soft tissue coverage after surgery was expected. Taking these points into account, and clearly explaining to the patient’s parents the expected necessity of further surgery and possible complications that could lead to amputation, the authors decided to pursue a limb-salvage solution.

The choice of a composite solution depended on different evaluations. A modular prosthesis of such a small size was not available. Moreover, there were concerns about two possible complications: first of all, the intramedullary stem perforation could compromise the distal femoral physis [22]. At the same time, due to the rapid growth of very young children, a rapid widening of the femoral canal was expected, with consequent loosening of the prosthesis.

The patient’s fibula at the time of surgery had a 5 mm diameter. It is the authors’ experience, also confirmed by Muscolo et al. [22], that younger children usually have good osteointegration, mostly when there is a good contact surface between the graft and the host bone. The possibility of a vascularized fibula graft was considered, but was rejected as the first choice because of the high failure rate.

The size of an adult femur is not suitable for reconstructing a child’s femur, the diameter of the diaphysis and the femoral head being too wide. A skeletally mature humerus, however, has a size that is suitable for replacing segments of a child’s femur or tibia.

The proximal humerus can also be used to reconstruct a child’s proximal femur; indeed, it can support a small stem prosthesis and the residual capsule can be helpful for reattaching the gluteal muscles. However, the proximal epiphysis is quite big and may be much too bulky for very small patients, as in the presented case.

Furthermore, the anatomy of the distal humerus is more like that of the femur; the distal epiphysis can be shaped to reproduce the femur better than the proximal epiphysis. The cortex is stiff enough to support a prosthesis implant, and, if lateral ligaments are also available, they can be used to reattach the gluteal muscles.

After gaining access to the olecranon fossa and then to the medullary canal, the broaches can be easily manipulated to obtain a good fit to the distal humerus; a small endoprosthesis can be cemented in the allograft, obtaining good primary stability (Figure 2B).

In the presented case, no collateral ligament was present, so trans-bone stitches were used with a satisfactory result; this ensured quite normal walking. A good fusion was also reached; this could be due to the higher biological activity and remodeling in young bone but also to the diapasonal shape of the distal allograft, which increases the bone-to-bone contact area. In addition, the cortical strut, cemented in the allograft medullary canal and fitted in the patient’s residual femoral canal, contributed to the primary stability and underwent remodeling.

Moreover, the carbon fiber plate may facilitate osteointegration because it stabilizes the segments in a more elastic manner than a titanium plate does; its radiolucency makes it possible to monitor graft fusion, and should ensure safer postoperative radiotherapy, due to the lower level of artifacts, if this is necessary [23,24]. The aim of overlapping the proximal plate edge and femoral stem is to prevent stress fractures of the graft by improving the load distribution.

Unfortunately, this surgery must be considered as just a temporary solution. With the growth of the patient, the increase in diameter of the acetabulum, and the onset of bone shortening, future operations will be unavoidable. When the dimension of the segments allows, a custom-made expandable prosthesis could be considered to decrease any difference in leg length.

Fortunately, the distal epiphyseal plate was spared, maintaining its growth potential. This could allow for a residual medullary canal large enough to house the prosthesis stem, and spare the epiphyseal plate as well; moreover, considering the good level of remodeling present at one year after surgery, it would be desirable that part of the homograft could be used to host the prosthesis stem, obviously after cement and cortical strut removal.

Furthermore, the greatest lengthening allowed by tight soft tissues (1 cm) was provided during surgery in order to delay successive elongation. Moreover, the use of a graft to fit the prosthesis avoided the problem of loosening due to canal widening, as may occur when a prosthesis is inserted in a growing bone. The use of the graft also allowed the preservation of bone stock.

Unfortunately, the proximal growth cartilage was too close to the tumor to be spared, but the choice of an endoprosthetic replacement allows the acetabular triradiate cartilage to continue to grow, also receiving the correct mechanical stress from a spherical femoral head.

Obviously, this technique also presents possible disadvantages related to the use of a homograft; indeed, its osteointegration is quite limited to the contact area (a few centimeters) so there is a resorption risk. Moreover, a frequent complication is a fracture that can barely heal. This is the most frequent cause of revision.

Reconstruction surgery in very young patients presents another important challenge to bear in mind: their post-operative and rehabilitation care. In the presented case, the hip had to be protected against possible dislocation as well as the graft-to-host junction, to allow graft fusion. It was therefore necessary to prevent the infant’s movement and weight-bearing by using a cast.

The choice of postoperative rehabilitation steps could greatly influence the functional result; indeed, precocious mobilization and weight-bearing could increase the risk of dislocation, but prolonged immobilization could cause excessive muscular fibrosis and weakness.

Obviously, the correct equilibrium must be found, also taking into consideration the young patient’s cooperability.

The present paper presents several limitations, particularly because it is a case report. However, our findings need to be confirmed in several further cases before they can be considered valid. In addition, more studies with long-term follow-up will be necessary to determine how to resolve problems related to patients’ growth and future prosthesis implants.

## Supplementary Materials

The following are available online at https://www.mdpi.com/article/10.3390/children8060442/s1: Video S1: A new proximal femur reconstruction technique after bone tumor resection in a very small patient: an exemplificative case.
